# Modulating the Biologic Activity of Mesenteric Lymph after Traumatic Shock Decreases Systemic Inflammation and End Organ Injury

**DOI:** 10.1371/journal.pone.0168322

**Published:** 2016-12-15

**Authors:** Simone Langness, Todd W. Costantini, Koji Morishita, Brian P. Eliceiri, Raul Coimbra

**Affiliations:** 1 Division of Trauma, Surgical Critical Care, Burns and Acute Care Surgery, Department of Surgery, University of California, San Diego Health Sciences, San Diego, California, United States of America; 2 Division of Acute Critical Care and Disaster Medicine, Tokyo Medical and Dental University, Tokyo, Japan; USF Health Morsani College of Medicine, UNITED STATES

## Abstract

**Introduction:**

Trauma/hemorrhagic shock (T/HS) causes the release of pro-inflammatory mediators into the mesenteric lymph (ML), triggering a systemic inflammatory response and acute lung injury (ALI). Direct and pharmacologic vagal nerve stimulation prevents gut barrier failure and alters the biologic activity of ML after injury. We hypothesize that treatment with a pharmacologic vagal agonist after T/HS would attenuate the biologic activity of ML and prevent ALI.

**Methods:**

ML was collected from male Sprague-Dawley rats after T/HS, trauma-sham shock (T/SS) or T/HS with administration of the pharmacologic vagal agonist CPSI-121. ML samples from each experimental group were injected into naïve mice to assess biologic activity. Blood samples were analyzed for changes in STAT3 phosphorylation (pSTAT3). Lung injury was characterized by histology, permeability and immune cell recruitment.

**Results:**

T/HS lymph injected in naïve mice caused a systemic inflammatory response characterized by hypotension and increased circulating monocyte pSTAT3 activity. Injection of T/HS lymph also resulted in ALI, confirmed by histology, lung permeability and increased recruitment of pulmonary macrophages and neutrophils to lung parenchyma. CPSI-121 attenuated T/HS lymph-induced systemic inflammatory response and ALI with stable hemodynamics and similar monocyte pSTAT3 levels, lung histology, lung permeability and lung immune cell recruitment compared to animals injected with lymph from T/SS.

**Conclusion:**

Treatment with CPSI-121 after T/HS attenuated the biologic activity of the ML and decreased ALI. Given the superior clinical feasibility of utilizing a pharmacologic approach to vagal nerve stimulation, CPSI-121 is a potential treatment strategy to limit end organ dysfunction after injury.

## Introduction

The gut plays a pivotal role in the pathogenesis of the systemic inflammatory response syndrome (SIRS) and the development of end organ dysfunction after trauma/hemorrhagic shock (T/HS) [1–3]. Following T/HS, splanchnic perfusion is decreased in order to preserve central circulation [4, 5]. This severe vasoconstriction can result in an ischemic/reperfusion injury to the gut, compromising gut barrier integrity and allowing for the translocation of bacteria and antigens [6, 7]. This “translocation” was long held as the inciting event in the pathogenesis of SIRS, however, recent studies have put this theory into question.

Moore et al. sampled portal vein from trauma patients during exploratory laparotomy and failed to identify bacteria or endotoxins in patients who ultimately developed multiple organ dysfunction syndrome (MODS) [8]. Newer theories have focused on the mesenteric lymph (ML) as the inciting source for the development of SIRS [2, 9]. These theories propose that pro-inflammatory mediators are produced in the gut after trauma, which can be released into the systemic circulation via the ML to cause and propagate the systemic inflammatory response [2, 10, 11].

While the lymph-derived pro-inflammatory mediators that cause SIRS have yet to be completely characterized [12–14], ample evidence exists to demonstrate their biologic activity. In vitro application of shock-derived ML causes endothelial cell dysfunction [15–17], neutrophil activation [17–19] and red blood cell deformation [10, 20]. In-vivo studies have shown that shock-derived ML contributes to the development of acute lung injury (ALI) [21–23] and cardiac dysfunction [24, 25] and that these effects are abrogated with mesenteric duct ligation prior to injury [16, 26].

Vagal nerve stimulation (VNS) augments the cholinergic anti-inflammatory reflex [27] and limits the inflammatory response following injury [28–30]. The mechanism by which VNS dampens inflammation is multifactorial and includes decreasing the inflammatory potential of ML following injury [31, 32]. Previous work in our lab has demonstrated that CPSI-121, a pharmacologic vagal agonist, is capable of attenuating the biologic activity of ML [33]. We aimed to determine if ML from CPSI-121-treated animals would limit SIRS and the development of end organ dysfunction following T/HS.

## Material and Methods

### T/HS Model

Male Sprague-Dawley rats weighing 280–300 grams (Harlan Laboratories, Placentia, CA) were anesthetized with ketamine (75 mg/kg; Fort Dodge Animal Health, Fort Dodge, IA) and xylazine (10 mg/kg; Sigma Chemical, St. Louis, MO), and the left femoral artery and vein were cannulated with a polyethylene tube (PE-50). Male rats were used to minimize the confounding effects sex hormones may have on the inflammatory process after injury [34, 35]. The mean arterial pressure (MAP) was continuously monitored using the femoral arterial catheter (Philips V24/26, Andover, MA) and body temperature was maintained at 37^o^ C with a warming pad. Trauma/sham shock (T/SS) was induced by performing a right medial visceral rotation through a midline laparotomy incision [33]. Hemorrhagic shock following trauma (T/HS) was induced via withdrawal of blood from the femoral vein catheter until the MAP was reduced to 35 mmHg and maintained for 60 minutes [21]. At the end of T/HS, animals were resuscitated with shed blood + two times shed blood volume in normal saline for 2 hours (Baxter, Deerfield, IL). A separate cohort of animals was treated with CPSI-121 (Ferring, San Diego, CA) diluted in sterile water immediately after induction of T/HS. CPSI-121 was administered at 1 mg/kg intravenously based on our previous experiments demonstrating gut barrier protection after injury using this dose [36]. N = 4 for all experimental groups.

### Collection of ML

The mesenteric duct was exposed and cannulated with a polyethylene tube (PE-50) prior to T/SS or T/HS [37]. ML was collected on ice during the T/SS or T/HS phase (60 minutes) and centrifuged at 2,000 rpm for 5 minutes at -4°C. The ML supernatant was stored at -80°C for future use.

### ML Infusion

Lymph samples from T/SS, T/HS and T/HS + CPSI-121 injuries were thawed and infused into naïve C57BL/6 male mice (N = 4 for all experimental groups) via a heparinized (0.1 U/mL) internal jugular cannula (PE-10) at a rate of 1 mL/kg/hr for 3 hours [32]. Sham animals were infused with heparinized saline only under the same conditions.

### Monocyte Signal Transducer and Activator of Transcription 3 (STAT3) Phosphorylation (pSTAT3)

Prior to lymph infusion, a heparinized catheter was placed in the femoral artery for continuous blood pressure monitoring (Philips V24/26, Andover, MA). 100 uL of blood was withdrawn prior to and immediately following lymph infusion. Whole blood samples were lysed and fixed with Lyse/Fix Buffer (BD Phosflow, Cat 548049, NJ) for 15 minutes at room temperature. After washing, leukocytes were permeabilized with Perm/Wash Buffer (BD Phosflow, Cat 554723, NJ) for 15 minutes and then incubated with primary PE-labeled anti-pSTAT3 (BD Biosciences, Cat 61258, 1:50) in FACS buffer. Flow cytometry was performed with a Becton Dickinson FACS Calibur. Monocytes were identified based on characteristic forward and side scatter as previously described [38]. Median florescence of monocyte pSTAT3 was analyzed for fold change after lymph infusion.

### Lung Tissue Collection

Murine lungs are comprised of four right lobes and one left lobe [39]. This finding can be exploited to perform various lung assays per animal. After the completion of lymph infusion, animals were sacrificed with cervical dislocation. A cardiopulmonectomy was performed and the left and right superior lobes were ligated at the bronchus with silk ties. After ligation, the right atrium was injected with 3 mL of cold PBS to flush the pulmonary vasculature of the remaining three right lung lobes. The middle superior lobe was ligated for lung histology and the middle inferior lobe was ligated for flow cytometry assay. The trachea was then isolated in order to inflate the final right inferior lobe with 4% paraformaldehyde for immunohistochemistry.

### Lung Permeability

Mice were injected with Evan’s Blue Dye (EBD) (30 mg/kg in PBS) via the femoral arterial catheter 30 minutes prior to the end of the 3 hour lymph infusion period as previously described [40]. The left lobe was weighed at the time of harvest and 24 hours later. The ratio of weights were compared to determine the wet:dry measurement. The right superior lobe was placed in formamide after ligation for 24 hours to collect extravasated EBD [41]. The absorbance of EBD/formamide solution (620 nm) was measured with a spectrometer and compared against known EBD dilutions.

### Lung Histology

The right middle superior lobe was fixed in formalin and embedded in paraffin. Sections of paraffin were stained with hematoxylin and eosin by the University of California, San Diego Histology Core Services (*n* = 3 mice per experimental condition). Investigator blinded to the experimental conditions then scored the lung sections according to the pulmonary injury scoring system previously described by our laboratory [42]. Briefly, lung sections are rated from 0 (normal) to 3 (severe) based on degree of intra-alveolar hemorrhage, pulmonary congestion, edema and inflammatory cell infiltration to yield a maximum possible score of 12. Lung injury scores were averaged for each experimental condition.

### Immunohistochemistry

Inflated lobes were fixed in 0.1 mol/L PBS containing 4% paraformaldehyde at room temperature for 30 minutes and mounted in OCT. 4 μm slices of lung were sectioned and fixed on glass slides. Sections were then washed with PBS prior to blocking for 30 minutes with 3% bovine serum albumin (BSA, Sigma) and incubated overnight with primary antibody (1:200). Primary antibodies included goat anti-MPO and goat anti-CD68 (Abcore) on separate slides. Sections were then treated with the secondary antibody Alexa Fluor 488 (chicken anti-goat IgG, Invitrogen, Waltham, MA) and Alexa Fluor 546 (rat anti-goat IgG, Invitrogen, Waltham, MA). Antibodies were buffered in 1% BSA for 1 hours at room temperature after washing with PBS (pH 7.4) for 5 minutes. Slow Fade (Invitrogen) was added prior to placement of cover slides. Images were obtained using an Olympus Fluoview laser scanning confocal microscope with exposure-matched settings at 20x and 40x magnification.

### Flow Cytometry

After ligation, lung tissue from the right middle inferior lobe was minced and incubated in the enzyme solution collagenase A/dispase II at 37°C for 20 minutes to isolated lung cells. The lung tissue was then passed through a 70 um filter and digestion was quenched with 5% fetal bovine serum solution. The cells were stained with anti-mouse monoclonal antibodies including PE Cy7-labeled anti-CD11c (HL3; BD Biosciences, San Jose, CA), APC Cy7-labeled anti-CD11b (M1/70; BD Biosciences), and APC-labeled anti-MHC II (M5/114.15.2; eBioscience, San Diego, CA). Mouse pulmonary macrophages were identified by techniques described by Vermaelen and Pauwels [43]. In brief, digested cells were first gated for CD11c and macrophages were identified based on high autofluorescence.

### Animals

This study was carried out in strict accordance with the recommendations in the Guide for the Care and Use of Laboratory Animals of the National Institutes of Health. The protocol was approved by the Institutional Animal Care and Use Committee (IACUC) of the University of California, San Diego (Permit Number: S13020). All surgeries were performed under either ketamine/xylazine or 1.5% inhaled isoflurane and all animals were monitored closely for pain, suffering, or moribund appearance per our IACUC approved protocol. While our protocol called for euthanasia if animals became moribund, we did not have any animals that displayed these signs and therefore did not have to euthanize any animals prior to the end of the experimental protocol.

### Statistical Analysis

Data are presented as mean value ± the standard error of the mean (SEM). Comparison between groups (experimental versus sham and experimental versus T/HS + CPSI-121) was made with a 2-tailed unpaired student t-test. P values of ≤0.05 were considered statistically significant.

## Results

### T/HS derived Lymph Produces a Systemic Inflammatory Response

**Fig 1:**
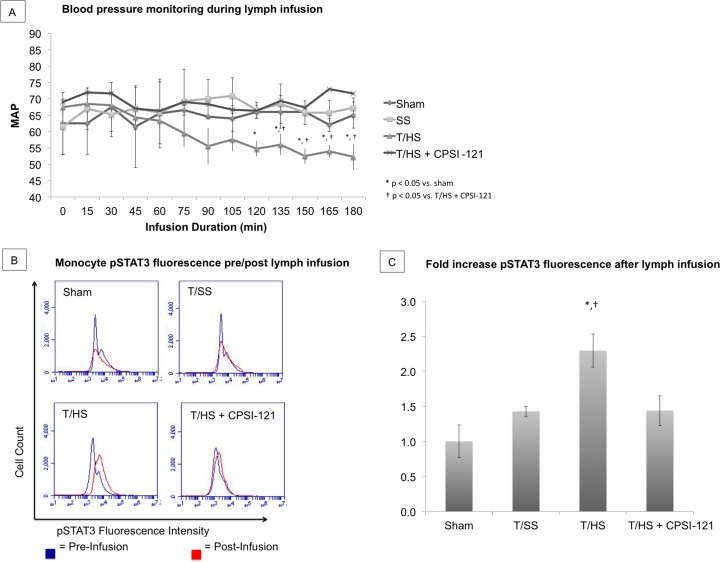
CPSI-121 prevents the systemic inflammatory response to T/HS. Mean arterial pressure ranged from 52–55 mmHg throughout the final hour of T/HS lymph infusion, which was statistically lower than MAP of sham mice or mice subjected to T/SS (64–68 and 66–69 mmHg, respectively, p<0.05) (A). T/HS + CPSI-121 derived lymph maintained MAP throughout lymph infusion with MAP readings similar to sham and T/SS (66-73mmHg, p = 0.79 versus sham). Flow cytometry histogram comparing monocyte STAT3 phosphorylation (pSTAT3) before (blue) and after lymph infusion (red) (B). T/HS lymph resulted in a 2.3 ± 0.47 fold increase in pSTAT3 fluorescence, which was significantly higher than the pSTAT3 fold changes after sham or T/SS lymph infusion (1.0 ± 0.31 and 1.43 ± 0.12, respectively, p<0.05). T/HS + CPSI-121 lymph resulted in 1.44 ± 0.31 fold increase in pSTAT3 fluorescence, which was not statistically different from sham (p = 0.102) (C).

Naïve mice infused with T/HS-derived lymph developed SIRS as evidenced by systemic hypotension and circulating monocyte activation (Fig 1A). Systemic hypotension occurred during the final hour of infusion in animals subjected to T/HS with MAP of 52–55 mmHg. No hemodynamic alterations occurred during the final hour of ML infusion from sham, T/SS or T/HS + CPSI-121 animals (MAP 65–69, 62–67 and 66–73 mmHg, respectively).

Circulating monocytes were identified on flow cytometry and the quantity of pSTAT3, an early marker of systemic inflammation [44], was compared before and after lymph infusion (Fig 1B and 1C). There was, on average, a 2.3 ± 0.47 fold increase in pSTAT3 expression after T/HS lymph infusion. By comparison, average fold increase in pSTAT3 expression after lymph infusion in sham and T/SS were 1.0 ± 0.31 and 1.43 ± 0.12, respectively. pSTAT3 elevation suggests increased activation of circulating monocytes after T/HS lymph infusion. CPSI-121 decreased the biologic activity of T/HS-derived ML and prevented the development of SIRS with hemodynamics and monocyte pSTAT3 expression similar to T/SS (1.44 ± 0.31 average fold change after lymph infusion).

### CPSI-121 attenuates the biologic activity of T/HS derived ML and limits the development of ALI

**Fig 2:**
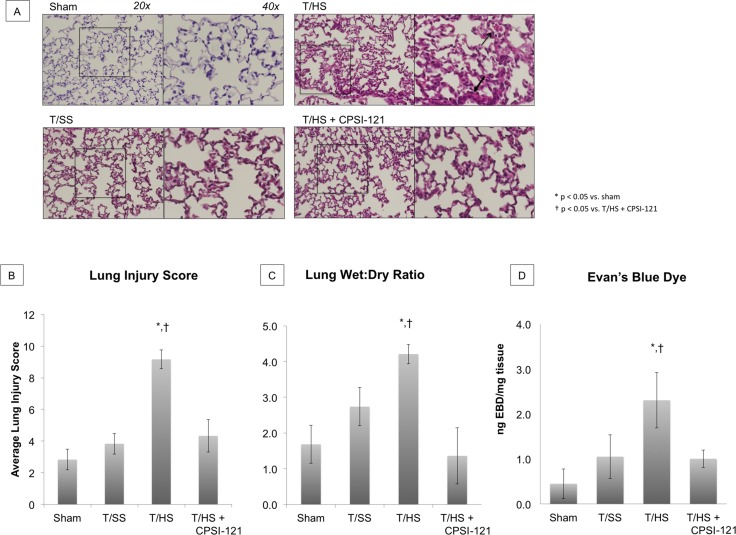
Acute Lung Injury Develops After T/HS Lymph Infusion and Is Attenuated by CPSI-121. Acute lung injury (ALI) is present on lung histology after T/HS lymph infusion as demonstrated by alveolar hemorrhage (open arrow) and thickened hyaline membrane (closed arrow) (A). Histology from sham animals had normal histologic features, with thin alveolar walls free from cellular infiltrate. T/SS and T/HS + CPSI-121 infusion resulted in similar findings on histology with mild airway edema and cellular infiltration compared to sham, but attenuated compared to T/HS. Pulmonary injury score was significantly higher after T/HS lymph infusion (9.17 ± 0.6) compared to sham (2.83 ± 0.65, p = 0.0001), T/SS (3.83 ± 0.65, p = 0.0001) or T/HS + CPSI-121 (4.33 ± 0.6, p = 0.0022) lymph infusion (B). Lung permeability, another marker of ALI, was significantly elevated in animals subjected to T/HS derived lymph compared to animals injected with sham, T/SS or T/HS + CPSI-121 derived lymph. Average wet:dry ratio was 4.21 ± 0.271 in the T/HS group compared to 1.683 ± 0.531, 2.738 ± 0.533 and 1.362 ± 0.786 in the sham, T/SS and T/HS groups, respectively (C). Similarly, Evan’s Blue Dye absorbance was significance higher in the T/HS group (2.305 ± 0.69) compared to sham (0.449 ± 0.33), T/SS (1.051 ± 0.49) and T/HS + CPSI-121 (1.004 0078 0.136) (D).

ALI is characterized by increased lung permeability and cellular infiltrate [45]. T/HS-derived lymph resulted in ALI with characteristic features of alveolar hemorrhage and airway edema on histology and increased lung permeability from wet-to-dry ratio and EBD absorption (Fig 2A–2D). T/SS and T/HS + CPSI-121 derived lymph produced mild airway edema on histology but had similar lung permeability to sham.

### CPSI-121 decreases T/HS lymph-induced inflammatory cell recruitment to the lung

**Fig 3:**
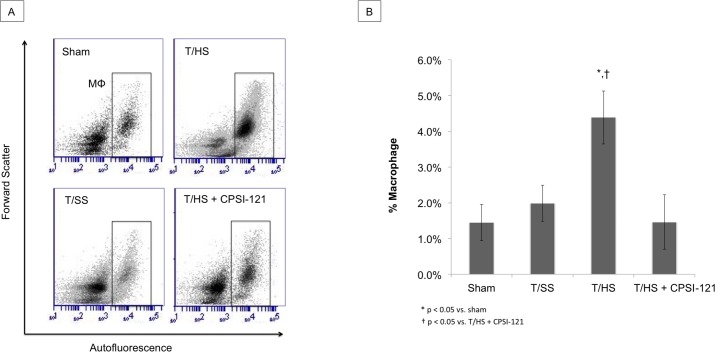
Lung Macrophages Increase in Number Following T/HS Lymph Infusion. T/HS lymph infusion resulted in increased amount of pulmonary macrophages present on flow cytometry (4.39% ± 0.75) compared to sham (1.41% ± 0.49, p = 0.026) and T/SS infusion (1.99% ± 0.51, p = 0.037) (A and B). T/HS + CPSI-121 lymph attenuated the pulmonary macrophage increase with quantity similar to T/SS (1.46% ± 0.27).

**Fig 4:**
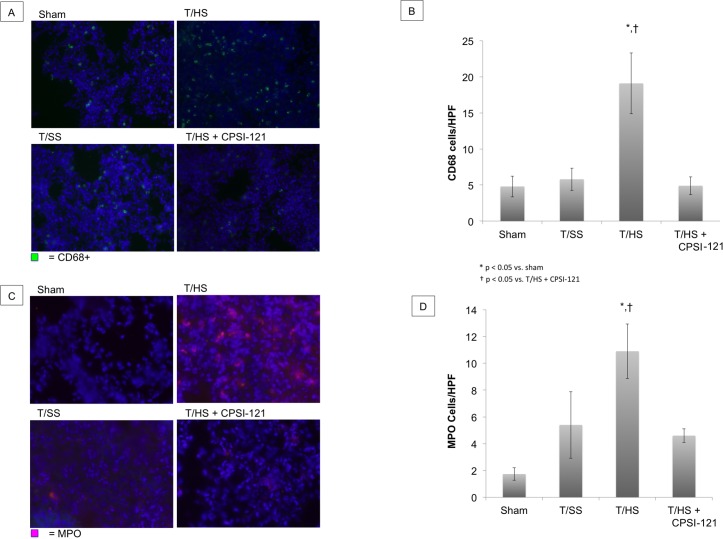
Immune Cell Infiltration of Lungs Following T/HS Lymph Infusion. An increase in lung macrophages was present on immunohistochemistry (IHC) following T/HS lymph infusion as demonstrated by CD68 staining (A). T/HS lymph infusion resulted in an average of 19.1 CD68+ cells/high power field (HPF) compared to 4.8 (p = 0.0329) and 5.8 (p = 0.0418) cells/HPF in sham and T/SS, respectively (B). T/HS + CPSI-121 lymph had similar CD68+ cells/HPF to T/SS (4.9). Neutrophils were also present in increased numbers on IHC in lung samples followings T/HS lymph infusion (10.9 MPO+ cells/HPF) (C) compared to sham (1.7 MPO+ cells/HPF, p = 0.0117) (D). Neutrophil numbers were also increased in T/HS lymph infusion compared to T/HS + CPSI-121 (4.6 MPO+ cells/HPF, p = 0.0139).

Pulmonary macrophages were present at higher concentrations after T/HS lymph infusion compared to sham on flow cytometry (4.39% ± 0.75 versus 1.41% ± 0.49, p = 0.026) (Fig 3). Macrophage flow cytometry findings were correlated with IHC staining for CD68, a macrophage marker (Fig 4A and 4B). Neutrophil counts were also elevated after T/HS lymph infusion with an average of 10.9 cells/HPF compared to 1.7 cells/HPF in sham infusions (p = 0.01) (Fig 4C and 4D). T/SS lymph infusion did not result in a statistically significant increase in macrophage and neutrophil levels. T/HS + CPSI-121 attenuated the T/HS-induced increase in immune cells in the lungs with macrophage and neutrophil counts similar to T/SS lymph.

## Discussion

The systemic inflammatory response that occurs after trauma results in damage to distant organs, most notably the lungs as seen in ALI or acute respiratory distress syndrome [46]. Ongoing or uncontrolled systemic inflammation can ultimately result in the development of multiple organ dysfunction syndrome (MODS) [47, 48], which is a major cause of mortality following trauma [49]. The pathogenesis of SIRS is incompletely understood at this time, but increasing evidence points to the gut and mesenteric lymph as key mediators [1, 50].

Our findings support the gut-lymph hypothesis of SIRS as lymph derived from T/HS animals caused SIRS in naïve animals whereas lymph derived from T/SS failed to do so. SIRS is thought to result from the activation of multiple leukocyte pro-inflammatory genes through complex signaling mechanisms. STAT proteins are a family of transcription factors induced by cytokines and growth factors. STAT3 is present in a variety of cell types and is increasingly being recognized for its role in the inflammatory response [44, 51, 52]. In airway epithelial cells, LPS induces strong STAT3 activation, which correlates with an increase in the pro-inflammatory cytokines TNF-alpha [53]. Moreover, STAT3 phosphorylation is required for monocyte to macrophage differentiation in atherosclerotic plaques and STAT3 inhibition results in a decrease in pro-inflammatory genes [54]. Gaudilliere et al. found that STAT3 was a marker of early systemic inflammation by demonstrating increased activation in circulating CD14+ monocytes following major surgery. Additionally, he found higher levels of pSTAT3 correlated with longer postoperative recovery [44]. We demonstrate increased pSTAT3 in circulating monocytes following T/HS lymph infusion compared to T/SS lymph infusion. Furthermore, infusion of T/HS lymph also produced systemic hypotension, further supporting a SIRS response in T/HS lymph treated animals [55, 56].

T/HS lymph also caused ALI in naïve animals compared to minimal lung pathology seen in animals subjected to T/SS lymph. ALI, characterized by hypoxia, edema and pulmonary infiltrates, contributes to the high mortality associated with MODS [45, 57]. Pulmonary edema is thought to develop secondarily to increased lung vascular permeability as well as immune cell activation and infiltration [58–60]. We found increased vascular permeability in lung samples of animals injected with T/HS lymph on both EBD and wet-to-dry assays. Additionally, these lungs samples had an increased quantity of macrophages and neutrophils on both flow cytometry and IHC, suggesting an immune cell infiltration. The ability of T/HS derived lymph to produce ALI is in line with previous work in in-vivo models showing similar results [11, 21, 22].

In our study, treatment with a pharmacologic vagal agonist at the time of injury limited the development of SIRS and ALI. These findings are in agreement with previous work by our lab and others, where VNS is capable of altering the biologic activity of ML [32, 33, 61]. While the exact mechanism has yet to be determined, VNS appears to alter the inflammatory response of resident gut macrophages as one potential mechanism for its anti-inflammatory effect [62]. VNS may inhibit the release of pro-inflammatory signals from resident macrophages, which then decreases the inflammatory signals present in ML. Another potential mechanism through which VNS may limit systemic inflammation is by altering the cellular composition of ML. Our lab has previously demonstrated T/HS results in a decrease in CD103+ MHC-II+ dendritic cells in ML [31]. Resident dendritic cells play an important role in regulating inflammation as they process antigens, which may be present after epithelial barrier breakdown, and alter the balance of T regulatory and T effector cells in the mesenteric lymph nodes. VNS prevents the depletion of these cells dendritic cells and pushes the inflammatory balance toward the tolerating T regulatory cell type. Further work in understanding which specific components of ML invoke biologically activity will aid in determining the mechanism by which VNS is able to exert its protective effect.

Currently, VNS can be applied though direct current or through pharmacologic strategies. CPSI-121, a guanylhydrazone-derived compound, has been shown to result in efferent vagal fiber activation after systemic administration and thus can be used to provide VNS through pharmacologic means [33]. While the exact therapeutic window is unknown, VNS appears to have the greatest benefit 1–2 hours following injury [10, 63]. Given the greater clinically feasibility of administering a pharmacologic agent over direct, electrical stimulation of the vagus nerve, pharmacologic VNS is an attractive option that may yield higher therapeutic potential. Findings from this series of experiments represent an important advance in our understanding of the ability of the vagus nerve to alter SIRS after injury. Previous work has demonstrated that CPSI-121 is protective after injury, in part due to restoration of gut barrier function [36]. In this study, we add further evidence for the protective effects of CPSI-121 through its ability to modulate the biologic activity of ML, which in turn, limits distant organ injury. These results highlight the promising potential of this pharmacologic vagal agonist in limiting intestinal and systemic inflammation after severe injury.

## Conclusions

T/HS results in biologically active ML that is injurious to distant organs. The pharmacologic vagal nerve stimulant CPSI-121 attenuates the biological activity of ML early after T/HS, decreasing both the systemic inflammatory response and the development of ALI. As such, CPSI-121 is a potential treatment strategy to limit end organ dysfunction after injury.
